# Diarrhoeal disease surveillance in Papua New Guinea: findings and challenges

**DOI:** 10.5365/wpsar.2018.9.2.006

**Published:** 2020-03-30

**Authors:** Mohammad Yazid Abdad, Kevin W Soli, Bang Pham, Grace Bande, Tobias Maure, Marinjo Jonduo, Debbie Kisa, Glennis Rai, Suparat Phuanukoonnon, Peter M Siba, Paul F Horwood, Andrew R Greenhill

**Affiliations:** a Papua New Guinea Institute of Medical Research, Goroka, Papua New Guinea.; b National Centre for Infectious Diseases, Singapore.; c Institute of Infectious Diseases and Epidemiology, Tan Tock Seng Hospital, Singapore.; d United States Centers for Disease Control and Prevention, Port Moresby, Papua New Guinea.; e College of Public Health, Medical and Veterinary Sciences, James Cook University, Townsville, Australia.; f School of Health and Life Sciences, Federation University Australia, Victoria, Australia.; * Corresponding author.

## Abstract

Diarrhoeal diseases are among the leading causes of morbidity and mortality in the Western Pacific Region. However, data on the major causes of infectious diarrhoea are limited in many countries within the Region, including Papua New Guinea. In 2013–2014, we conducted surveillance for acute diarrhoeal illness in four provinces in Papua New Guinea. One rural health clinic from each province participated in the surveillance activity. Samples were sent to central laboratories and batch analysed for bacterial and viral gastrointestinal pathogens that are commonly associated with diarrhoea. Across the four sites, the most commonly detected pathogens were *Shigella* spp., *Campylobacter* spp. and rotavirus. In this paper, we report the results of the surveillance activity and the challenges that we faced. The lessons learnt may be applicable to other parts of the Region with a similar socioeconomic status.

Diarrhoeal diseases are among the leading causes of morbidity and mortality globally. They are the sixth greatest contributor to disability-adjusted life-years across all age groups. However, the burden of diarrhoea is most significant in children, particularly under the age of 5 years. For individuals aged one month to 5 years, diarrhoea is the second leading cause of disability-adjusted life-years. (*1*) Diseases related to unsafe water, poor sanitation and lack of hygiene are some of the most common causes of disease outbreaks and death among the poor in developing countries.

In Papua New Guinea, it is estimated that only 40% of people have access to improved water supply and less than 20% have access to adequate sanitation, (*2*) one of the lowest rates in the world. Consequently, enteric infectious diseases remain a serious health concern as one of the major causes of hospitalization and death in the country. (*3*) Despite this, little is known about the etiology of diarrhoea in Papua New Guinea because of a lack of laboratory facilities and human resources to support the necessary microbiologic analysis. An overview of the current knowledge can be accessed in an editorial (*3*) and a review (*4*) published in 2013. Identification of pathogens is essential for improved clinical management and effective prevention strategies.

When we began surveillance in 2013, there was no monitoring of the etiology of diarrhoeal disease in Papua New Guinea. We established diarrhoeal disease surveillance for acute diarrhoeal illness at four health clinics in different regions of Papua New Guinea. The etiology of diarrhoeal illness and the challenges of establishing and maintaining ongoing disease surveillance in Papua New Guinea are discussed.

## Methods

### Site selection

**Figure 1:**
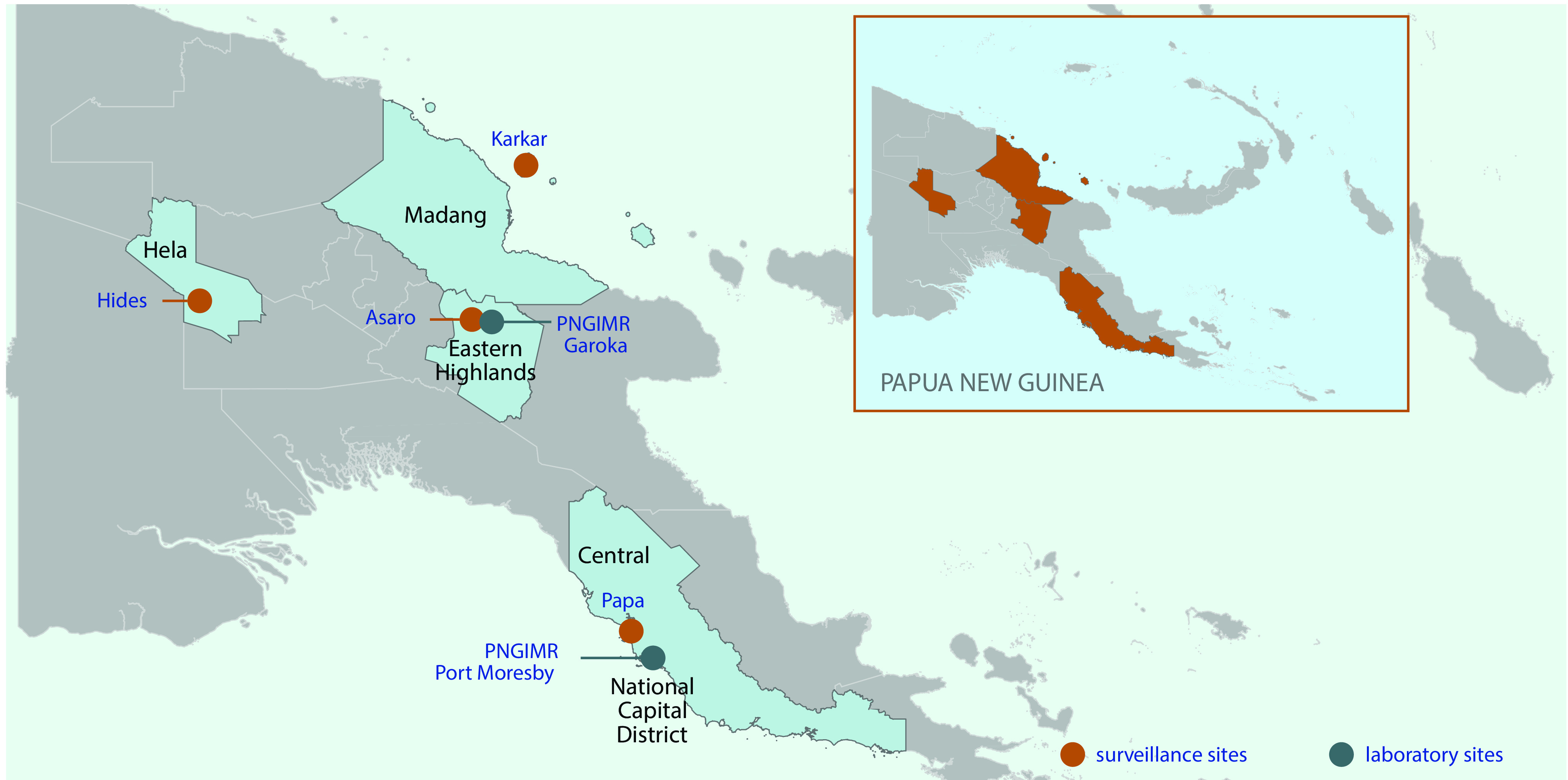
**Map of Papua New Guinea showing surveillance sites and laboratory sites**

Surveillance sites were established in four of the 22 provinces of Papua New Guinea, with each province represented by one rural health clinic. The surveillance locations were Malanda Clinic, Hela Province; Asaro, Eastern Highlands Province; Karkar Island, Madang Province; and Papa, Central Province (**Fig. 1**). The four surveillance sites were selected to be representative of the country. Papua New Guinea has a dispersed population, with over 70% of the population living in rural and regional areas. Thus, the four selected sites were non-urban, though two sites (Papa and Asaro) were readily accessible to major urban areas (Port Moresby and Goroka, respectively). The surveillance was conducted as part of a public–private partnership between a government-funded medical research institute and a private enterprise, with the intention to conduct demographic surveillance over 4 years. Thus, some requests of the industry sponsor of this study were considered, including setting up surveillance in or near areas of industry activity (Malanda Clinic and Papa sites). Patient samples were collected in 2013 and 2014.

[**insert** Figure 1]

### Case definition

We used the World Health Organization’s case definition for diarrhoea – the passing of three or more loose or watery stools in a 24-hour period. (*5*) We did not actively exclude people with chronic diarrhoea. Data were collected at the clinics using a specially designed case reporting form that included socio-demographic variables in addition to general clinical findings.

### Participants

Patients who presented to one of the participating health clinics with a diarrhoeal illness fitting the case definition were asked to provide a self-collected stool sample. A study nurse verbally described the implications, objectives and rationale of the surveillance activity to each potential participant. The study nurse also provided instructions on specimen collection that aimed to ensure personal hygiene for patients and sample integrity. Participation in the surveillance activity was voluntary, and verbal consent was obtained. Logistical constraints in the storage and transport of samples in this resource-limited setting necessitated batch testing of samples. Consequently, clinically relevant results could not be provided to the clinic or the patient.

### Sample processing and laboratory analysis

Samples were stored at 4 °C for less than 24 hours before their transport to the Papua New Guinea Institute of Medical Research laboratories (**Fig. 1**), where they were aliquoted and stored at –80 °C until analysis.

Nucleic acids were extracted from stool samples using the QIAamp DNA Stool Mini Kit (Qiagen, Hilden, Germany) and eluates were stored at –80 °C until batch testing. Molecular analysis using real-time polymerase chain reaction was conducted for a wide range of enteric pathogens. The bacterial pathogens targeted were *Shigella* spp., *Salmonella* spp., *Vibrio cholerae* (species level [*hylA*] and toxigenic strains [*ctxA*]), and *Campylobacter* spp. Several viral pathogens, including rotavirus, adenovirus 40/41, norovirus GI, norovirus GII, sapovirus and astrovirus, were also targeted. Detection methods were based on those previously described. (*6*)

### Ethics statement

This study was conducted as part of the Partnership in Health programme. The programme was approved by the Papua New Guinea Medical Research Advisory Committee (MRAC No. 10.17).

## Results

This surveillance was embedded in a broader demographic and health surveillance programme. (*7*) The total numbers of surveillance participants and diarrhoeal cases are provided in **Table 1**. The etiological agent of diarrhoea was investigated for 118 patients presenting to the participating clinics with diarrhoea (**Table 2**). The median age of patients presenting with diarrhoea was 19.8 months, with an age range of 1 month to 69 years (*n* = 81 patients with age data). Males represented 63.6% of the patients who presented with acute diarrhoea (*n* = 99 patients with sex data). An enteric pathogen was detected in 85.6% (*n* = 101) of the samples tested. *Shigella* spp. (38.1%), *Campylobacter* spp. (33.1%), rotavirus (20.3%) and norovirus genogroup GII (12.7%) were the pathogens most commonly detected. All other pathogens were detected at varying rates below 10%, including sapovirus (8.5%), adenovirus (5.9%), astrovirus (2.5%), norovirus genogroup GI (1.7%), *Salmonella* spp. (1.7%) and *V. cholerae* (1.7%). The two *V. cholerae*–positive samples were further investigated to determine if they were toxigenic strains, but both faecal samples were negative for the *ctxA* gene. Mixed infections were detected in 31.4% (*n* = 37) of diarrhoeal cases, including seven patients concurrently infected with three pathogens and two patients with four pathogens.

**Table 1:** Number of participants presenting to the primary health facilities where etiological surveillance was conducted (all) and those presenting with diarrhoea, 2013–2014^a,b^

-	Site				
	Papa	Malanda	Asaro	Karkar	Total
2013 diarrhoeal	73 (6.0%)	181 (20.0%)	72 (10.0%)	NA	**326 (11.4%)**
2013 all	1222	907	721	NA	**2850**
2014 diarrhoeal	494 (8.5%)	721 (10.1%)	242 (8.8%)	38 (1.6%)	**1495 (8.3%)**
2014 all	5800	7119	2748	2352	**18019**

NA: not available

^a^ The proportion of all participants presenting with the primary symptom of diarrhoea is provided in parenthesis.

^b^ Etiological analyses were conducted on approximately 5% of samples from patients presenting with diarrhoea in 2013–2014.

**Table 2:** Rates of detection of gastrointestinal pathogens commonly associated with diarrhoea from four surveillance sites in Papua New Guinea, 2013–2014

-	Site				
	Papa	Malanda	Asaro	Karkar	Total
Total samples	53	33	22	10	**118**
Rotavirus	10 (18.9)	6 (18.2)	4 (18.2)	4 (40.0)	**24 (20.3)**
Rorovirus GI	0 (0)	2 (6.1)	0 (0)	0 (0)	**2 (1.7)**
Norovirus GII	10 (18.9)	1 (3.0)	3 (13.6)	1 (10.0)	**15 (12.7)**
Adenovirus	2 (3.8)	1 (3.0)	1 (4.5)	3 (30.0)	**7 (5.9)**
Sapovirus	7 (13.2)	1 (3.0)	1 (4.5)	1 (10.0)	**10 (8.5)**
Astrovirus	1 (1.9)	0 (0)	2 (9.1)	0 (0)	**3 (2.5)**
*Shigella*	27 (50.9)	7 (21.2)	9 (40.9)	2 (20.0)	**45 (38.1)**
*Salmonella*	1 (1.9)	0 (0)	0 (0)	1 (10.0)	**2 (1.7)**
*Campylobacter*	10 (18.9)	21 (63.6)	6 (27.3)	2 (20.0)	**39 (33.1)**
*Vibrio cholerae*	2 (3.7)	0 (0)	0 (0)	0 (0)	**2 (1.7)**

## Discussion

Papua New Guinea is a tropical, developing country. It is prone to infectious disease outbreaks, as demonstrated by the maiden (and thus far only) outbreak of cholera in 2009–2011, which was the result of a single incursion. (*8*, *9*) The capacity for conducting outbreak investigations in Papua New Guinea is currently low. (*10*) Notwithstanding the need for increased laboratory capacity to rapidly confirm the etiology of outbreaks, there is also a distinct lack of baseline data on endemic disease in Papua New Guinea. Diarrhoeal illnesses can be caused by a wide range of viral, bacterial and protozoan infections, and it is difficult to ascertain the etiological agent based on clinical characteristics. The lack of knowledge of the epidemiology of diarrhoeal disease in Papua New Guinea negatively impacts the development and implementation of targeted control strategies.

In this study, *Shigella* spp., *Campylobacter* spp. and rotaviruses were the leading causes of diarrhoea at all four sites where samples were collected. These results are mostly consistent with previous investigations of the etiology of diarrhoea in Papua New Guinea. Soli et al. (*6*) detected *Shigella* (26.6%) and rotavirus (25.6%) as the leading causes of acute watery diarrhoea in hospitalized paediatric patients from the Eastern Highlands region of Papua New Guinea. Similarly, in a case-control study conducted approximately 30 years before our recent surveillance in the Eastern Highlands region, Howard et al. (*11*) detected rotavirus, *Shigella* spp. and *Campylobacter* spp. in 23%, 13% and 12% of cases, respectively. Additional studies have highlighted the importance of *Shigella* spp. in Papua New Guinea as the cause of an outbreak in a displaced population (*12*) and as a contributor to diarrhoea in both adults and children. (*13*)

One of the shortcomings in our understanding of infectious diseases in Papua New Guinea is our limited knowledge of the geographical distribution of many infectious agents. Through this surveillance, we gained insights into the etiology of diarrhoeal disease in geographically distinct locations in Papua New Guinea. To our knowledge, this is the first time that surveillance data containing diarrhoeal etiology have been collected concurrently from multiple geographical locations in Papua New Guinea. Despite the small sample sizes, our surveillance data provide preliminary evidence that pathogens such as *Shigella* spp., *Campylobacter* spp. and rotavirus may be significant contributors to gastrointestinal illness throughout the country. The corroboration of our data with previously obtained data strengthens our understanding of the major gastrointestinal pathogens associated with diarrhoea in Papua New Guinea.

Our surveillance system had several limitations. As with most infectious disease surveillance systems, the detection of a pathogen does not confirm that a specific pathogen is the cause of illness. This assumption is evidenced by our recent study where gastrointestinal pathogens were detected in asymptomatic participants in similar geographical locations in Papua New Guinea. (*14*) Moreover, the common detection of multiple pathogens in participants, a finding supported in our recent work, (*6*) makes it difficult to ascertain which pathogen, or combination of pathogens, is causing the presenting illness in these participants. The detection of recognized gastrointestinal pathogens is of public health importance irrespective of whether they cause diarrhoea: individuals carrying these pathogens are reservoirs of infection whether or not they are symptomatic. However, in symptomatic participants, the risk of pathogen dissemination increases because of increased frequency of defecation, high pathogen loads, and inadequate sanitation and hygiene.

In our surveillance system, we did not seek to detect protozoal pathogens, although parasites such as *Giardia lamblia* and *Entamoeba histolytica* have been detected in pregnant women in the highlands of Papua New Guinea in recent years. (*15*) Unfortunately, we could not include all potential etiological agents when conducting diarrhoeal disease surveillance, particularly in low-income settings.

In addition to the limitations inherent to many surveillance programmes, we encountered many logistical and funding challenges that affected our ability to establish ongoing and sustained surveillance. We initially selected four sites for ongoing surveillance. Site selection was determined largely by a programmatic health agenda, dictated by the industry partner. Establishing and maintaining surveillance activities at remote sites is challenging. The financial and human resource costs are considerable, and all organizations need to consider where their efforts in health care are best directed. We believe that in this context, a smaller number of surveillance sites with pre-existing support networks (including laboratory and sample storage infrastructure) would be more appropriate. The clinics selected in this study, like many regional and rural health clinics in Papua New Guinea, lacked the appropriate facilities for aseptic and culturally appropriate collection of stool samples. At three of the four clinics, we could not start stool sample collection until toilets were built or commissioned. All of the clinics lacked reliable running water, making proper handwashing after the passing and handling of stool samples difficult. Restricted access to electricity impacted sample storage (in fridges/freezers), which, in turn, influenced our approach to sampling. Opportunistic sampling was implemented because of the unavailability of suitable sample storage and infrequent opportunities to transport specimens.

Routine diagnosis of infectious diseases in Papua New Guinea is limited predominantly to diseases diagnosed from a small volume of blood using point-of-care tests (e.g. malaria, HIV). Health-care providers in Papua New Guinea infrequently request other types of clinical specimens used to diagnose disease, particularly stool samples, even when the diagnostic capacity exists. The infrequency of routine stool collection may have led to the reluctance of patients to provide stool samples, perhaps because of their unfamiliarity with the procedure or cultural discomfort. Further sociocultural studies are required to investigate these issues before future surveillance of gastrointestinal pathogens.

Transport of diagnostic samples to central laboratories was hindered by the low number of samples being received and the challenging logistics in Papua New Guinea. The road network is an impediment to health delivery as observed during the cholera outbreak in 2009–2011. (*16*) In some cases, poor roads made transport of consumables to the clinics and specimens to the laboratories difficult. No major logistical challenges impacted the transport of samples from the Asaro site to the nearby Goroka laboratory. However, the road connecting Goroka to Hides (location of Malanda Clinic) and Madang (a major town on the Papua New Guinea mainland near Karkar Island) is frequently impassable as a result of natural disasters, poor road maintenance and security threats. Samples from Papa were transported without difficulty to Port Moresby and processed in a recently established biomedical research laboratory in Port Moresby associated with the Papua New Guinea Institute of Medical Research and The University of Papua New Guinea.

Health facilities, including the study clinics, were closed at times for extended periods because of political unrest and violence during and following the 2012 election campaign. These disruptions negatively affected the establishment and continuation of surveillance. More importantly, but outside the scope of this work, they also negatively impacted the delivery of health services in times of increased need.

Despite the limitations and challenges associated with this surveillance, the relevance of the data should not be undervalued. We have furthered our knowledge of diarrhoeal pathogens circulating in Papua New Guinea. In doing so, we have identified significant potential challenges in establishing indicator-based surveillance. We are not suggesting that surveillance cannot, or should not, be conducted in Papua New Guinea. Event-based surveillance has been successfully implemented in other instances (*17*) and is an extremely useful tool for monitoring outbreaks. Indicator-based surveillance and event-based surveillance complement each other and are considered essential components of a single national surveillance system. (*18*) Attempts to establish indicator-based surveillance could benefit from an awareness of the challenges faced here.
